# Quantitative MALDI-MSI Workflow for Lipids with a LC-MS-Based Quality Control Pipeline

**DOI:** 10.1021/acsmeasuresciau.6c00083

**Published:** 2026-06-06

**Authors:** Felix-Levin Hormann, Sven Heiles

**Affiliations:** † 28371 Leibniz-Institut für Analytische Wissenschaften - ISAS - e.V., Otto-Hahn-Straße 6b, 44139 Dortmund, Germany; ‡ Lipidomics, Faculty of Chemistry, University of Duisburg-Essen, Universitätsstrasse 5, 45141 Essen, Germany

**Keywords:** mass spectrometry imaging, lipidomics, quantification, MALDI, lipid metabolism

## Abstract

Mass spectrometry imaging (MSI) has established itself as a major analytical method in the spatial analysis of biological tissue. Besides qualitative mapping of analyte distributions, recent developments aim to achieve quantification of target analytes. After successful spatial quantification of pharmaceuticals, the field is now shifting toward endogenous biomolecules such as lipids and metabolites. Here, we present a quantitative MSI (qMSI) workflow for endogenous lipids employing matrix-assisted laser desorption/ionization (MALDI) and stable isotope labeled standards (SILS) integrated into a quality control pipeline utilizing quantitative reversed-phase liquid chromatography tandem mass spectrometry (RP-LC-MS/MS). We thoroughly characterized the standard deposition of 13 lipid SILS with a focus on homogeneity and detection errors on mouse heart septa with intraday coefficient of variations (CVs) of ≤15%. Unlike RP-LC-MS/MS for which CV values increase mainly for lowly abundant lipids, variations of lipids in MALDI-MSI are affected by concentration as well as lipid class. Additionally, we compared lipid annotations based on MALDI-MSI and RP-LC-MS/MS and correlated these annotations between the methods based on quantification results. This method comparison revealed that most polar lipids such as PCs or SMs are captured in both MALDI-MSI and RP-LC-MS/MS, whereas less polar compounds such as TGs exhibit limited overlap. These results highlight that a determination of the SILS amounts and lipid annotations via RP-LC-MS/MS is necessary to counter the increase variability during quantitative MALDI-MSI. To demonstrate the analytical capabilities of the quantitative MALDI-MSI pipeline combined with RP-LC-MS/MS quality control steps, we analyzed mouse kidney from a two-hit heart failure with preserved ejection fraction model and achieved good comparability between RP-LC-MS/MS and MALDI-MSI quantitation results. Our data suggest increased ether lipid concentrations in obese mice kidney, likely to cope with increased oxidative stress induced by the progressing disease state of the mice.

## Graphical abstract

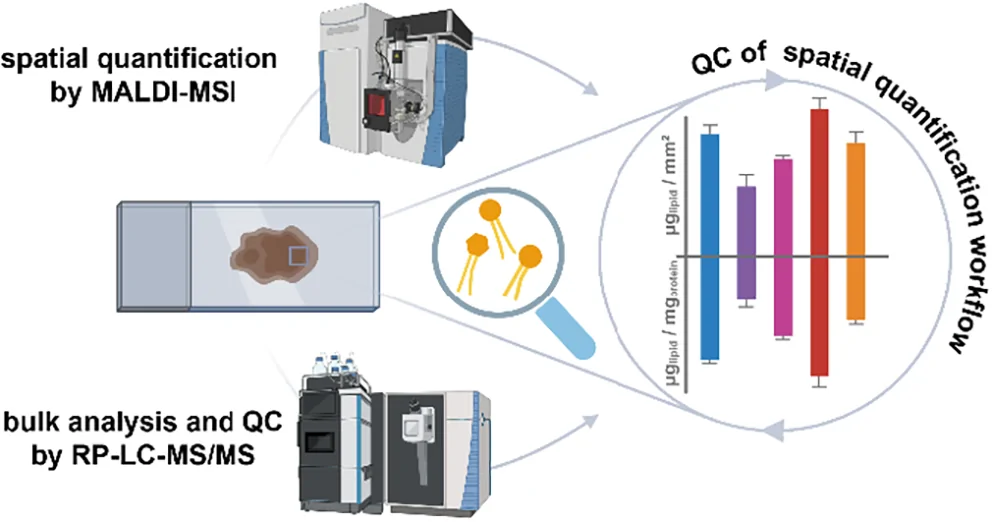

## Introduction

Mass spectrometry imaging is a valuable tool in the field of bioanalytics for the spatial analysis of metabolites, proteins, and drugs in tissue.^1,2^ It covers the macroscopic to the microscopic length scale, from whole body imaging of rodents^3–5^ down to the visualization of single cells.^6–9^ MSI of tissues is able to identify different histological and pathological regions thereby providing molecular signatures for diagnosis or revealing underlying biological events during disease progression.^10–12^ For example, Kaya et al. successfully applied multimodal MALDI-MSI with dual polarity lipid imaging and subsequent protein imaging to elucidate structural remodeling of infarcted myocardial tissue and interpret the connections between metabolite and protein changes.^13^ The most common ionization techniques include matrix-assisted laser desorption/ionization (MALDI) and desorption electrospray ionization (DESI)-MSI. Despite the widespread use in life sciences, MSI methods face technical shortcomings that complicate the transition from preclinical research to clinical practice.^1,14–16^ Most notably and despite recent efforts, accurate and reproducible absolute quantification of endogenous biomolecules remains challenging. Quantification, however, is required to probe reference values patient-by-patient to derive diagnostic and potentially therapeutic information. The major challenge for quantitation in MSI of endogenous compounds stems from tissue-specific ion suppression effects.^17–20^ This umbrella term describes influences by matrix properties, sample preparation, chemical background of the sample, and the interactions of analytes during the ionization process.^21,22^ As a result, the relative signal intensity of a specific analyte might not reflect its actual concentration in relation to all other analytes in tissue, preventing the comparison between tissue types without quantification. Therefore, SILS are required to enable quantitation with MSI methods by mitigating ion suppression effects.

To perform quantification and determine parameters such as limit of detection (LOD) and limit of quantification (LOQ) of a MSI method, recording of calibration curves in suitable tissue matrixes is the most accurate approach. This has been predominantly done in the field of drug quantification as summarized in a recent review of Tobias and Hummon.^23^ Construction of such calibration curves can be done by spotting different concentrations of the analyte onto the sample target, a control tissue, or by preparing mimetic tissue models with different concentrations spiked into tissue homogenates. Numerous examples for the spatial quantification of pharmaceuticals on different platforms have been reported.^24–34^ As an example, Hamm et al. have introduced a normalization factor termed tissue extinction coefficient to compensate for tissue-specific ion suppression effects in whole body imaging of rodents and applied it for the quantification of propranolol and olanzapine in mice after generating calibration curves next to the tissue sections.^32^ Sammour et al. performed a first clinical qMSI study on the uptake of imatinib in liver of cancer patients.^33^ Quantification was based on calibration curves spotted onto control liver tissue and a nonlinear regression. Barry et al. performed an extensive study on the use of mimetic tissue models for quantification of clozapine in rat liver, kidney, and brain alongside suggestions for data analysis and reporting of qMSI data.^34^ Also some seminal studies that used mimetic tissue models spiked with SILS for quantification of endogenous compounds have been reported.^35^ For example, Jadoul et al. quantified endogenous PCs in mouse brain sections based on a spiked brain homogenate model^35^ and calibration curves generated from different concentrations of PC standard sprayed onto brain sections.^36^ In a different approach, Davidová and Lanekoff introduced a standard addition-based method for quantitative nano DESI-MSI of metabolites in brain.^37^ However, the majority of recently published work on quantification of endogenous analytes focuses on single point calibration for quantification, using homogeneously applied SILS on the sample surface for normalization of target analytes with the signal of the corresponding SILS.^38–40^ Vandenbosh et al. proposed the use of a specifically composed standard mix of 13 lipid classes for application and normalization of lipid intensities.^38^ In a study focusing on metabolites, Wang et al. used ^13^C labeled yeast extracts for quantification of metabolites and lipids.^40^ As these internal standards experience similar ionization conditions as the analytes of interest, ion suppression effects can be minimized. If the amount of SILS applied onto the surface is exactly known, the SILS signal can be employed to quantitate endogenous compounds. However, the amount of SILS delivered onto the surface, if quantitation results are not to be affected by SILS losses during surface deposition, needs to be quality controlled with an independent method.

In this study, we present a quantitative MALDI-MSI workflow for endogenous lipids in which the amount of SILS and the annotations are systematically controlled by quantitative untargeted RP-LC-MS/MS. We establish and validate a pneumatic spraying protocol for homogeneous application of a SILS mix comprised of 13 individual lipid species with a quality control pipeline to determine the exact amounts of deposited SILS. Next, comprehensive analysis of mouse heart septa by both modalities has been performed and is compared between MALDI-MSI and RP-LC-MS/MS with regard to lipid annotations and measurement errors of quantification results. Last, we applied the presented multimodal workflow to analyze mouse kidney samples from the same biological set of mice, which have been fed with either a normal rodent diet (ND) or a high-fat diet combined with *N*_ω_-nitro-l-arginine methyl ester hydrochloride (l-NAME) administration. The latter is an established two-hit heart failure with preserved ejection fraction (HFpEF) mouse model, established by Schiattarella et al.^41^ These mice were additionally treated with nitro oleic acid (HFD-N) or a vehicle (HFD-V). Recently, Müller et al. published an in-depth analysis of the given mouse model and the potential therapeutic function of nitro oleic acid (NO2-OA) in the treatment of HFpEF.^42^

## Materials and Methods

### Materials

All chemicals are of HPLC-MS grade quality. Ammonium acetate (NH_4_Ac) was purchased from Sigma-Aldrich (Germany). EquiSplash SILS mix was purchased from Avanti Polar Lipids (USA). Ammonium format (NH_4_HCO_2_), formic acid (FA), and *tert*-butyl methyl ether (MTBE) were purchased from Merck (Germany). Water, methanol (MeOH), propan-2-ol (*i*-PrOH), and acetonitrile (ACN) were purchased from Biosolve (The Netherlands). 2′,5′-Dihydroxyacetophenon (DHAP) was purchased from Fisher Scientific (Germany).

### Animals

Male C57BL/6 N mice were purchased from Janvier Laboratories (Le Genest-Saint-Isle, France). They were housed for at least 1 week to acclimatize to laboratory conditions before starting any experimental procedure. All mice were kept in a 12:12 h inverse light cycle in ventilated cabinets that were maintained at 23 ± 2 °C and 40–60% humidity. Routine tests were performed to ensure that mice are pathogen-free according to the guidelines of the Federation of European Laboratory Animal Science Associations (FELASA). Mice had unrestricted access to water and food. For organ extraction, mice were analgised with buprenorphine (0.1 mg/kg BW) and anesthetized using isoflurane inhalation.

All animal studies were approved by the local Animal Care and Use Committees (Ministry for Environment, Agriculture, Conservation, and Consumer Protection of the State of North Rhine-Westphalia: State Agency for Nature, Environment, and Consumer Protection (LANUV), NRW, Germany) and followed guidelines from Directive 2010/63/EU of the European Parliament on the protection of animals used for scientific purposes.

Cardiometabolic syndrome was induced in male mice by HFD combined with the established eNOS inhibitor l-NAME, as described by Schiattarella et al.^41^ 4-week-old male mice were fed an HFD (60% fat, 20% protein, 20% carbohydrates; E15742–34; Ssniff, Germany) or standard rodent diet (ND group; V1534–703; Ssniff, Germany). l-NAME (0.5 g/L; no. N5751; Sigma-Aldrich) was supplied in the drinking water. The HFD + l-NAME and ND groups were fed for 11 weeks, and body weight was determined every week.

After 11 weeks, the HFD + l-NAME group was divided into two groups (vehicle or NO2-OA). Nitro oleic acid (50:50 mix of (E)-9- and 10-nitrooctadec-9-enoic acid; NO2-OA, was synthesized in-house^43^) and vehicle (poly­(ethylene glycol)/ethanol 90:10) were administered with ALZET mini-osmotic pumps (NO2-OA 8.17 mg/kg bw/day, model 2002, ALZET, Cupertino, CA, USA) for four weeks. After final investigations at 15 weeks, the organs were harvested and snap-frozen in liquid nitrogen.

### MALDI-MSI Sample Preparation

Samples were sectioned at −20 °C and 20 μm thickness with a microtome (CM1860, Leica, Germany) and stored under vacuum at −80 °C until further use. Sections were placed in a desiccator filled with silica gel at atmospheric pressure for 20 min to slowly thaw. The sample was placed in a home build pneumatic sprayer and coated with 100 μL at a flow rate of 10 μL/min of a EquiSplash SILS mix solution (33.3 μg/mL in MeOH/water (1:1, v/v)). The tissue was kept for 10 min in a desiccator to incubate the standard. DHAP matrix was applied in a commercial sublimation chamber (Chemglass Life Sciences, USA). In short, 300 μL of a 20 mg/mL DHAP solution in acetone was added to the bottom chamber. After evaporation of the solvent, the sample was attached to the cooling finger and the apparatus was assembled. The chamber was evacuated to 0.5 mbar and after 2 min the cooling finger was filled with an ice-water-NaCl mixture at −15 °C. After 4 min the sublimation chamber was lowered into a heated oil bath at 130 °C for an additional 4 min until all matrix crystals have been sublimated. Immediately after opening of the sublimation chamber, the sample was placed in a desiccator for 5 min. Each day, the standard application was controlled by coating a blank glass slide with the SILS mix in the described spraying method. The coated glass slide was kept in a desiccator for 10 min, washed three times with 150 μL MeOH, and the obtained solution was analyzed by RP-LC-MS/MS.

### MALDI-MSI Analysis

Measurements were performed on an AP-SMALDI^5^ AF ion source (TransMIT GmbH, Germany) coupled to an orbital trapping mass spectrometer (Q Exactive HF, Thermo Fisher Scientific, Germany) at a step size of 15 μm, a mass resolution of 240,000 at *m*/*z* 200, and a mass range of *m*/*z* 300–1200. For each tissue sample a region of minimum 5625 pixel was measured for statistical analysis. MALDI-MSI data were converted to imzml format using the RAW2IMZML converter (v1.8r3, TransMIT GmbH, Germany). Image generation, lipid identification, and export of peak intensities was carried out in LipostarMSI (version 2.0.1, Molecular Horizon srl, Italy). Identifications are based on MS^1^ and the LipidMAPS database. Lipid surface concentrations were calculated from mean peak intensities over all pixels of the investigated measurement area and multiplication by the surface concentration of the internal standard determined by the daily spray monitoring.

### Lipid Extraction

Lipids were extracted based on an adapted Matyash extraction protocol.^44^ In short, three tissue sections of 20 μm thickness were transferred into Eppendorf tubes and 200 μL ice cold 0.1% NH_4_Ac solution, 1 μL EquiSplash and six glass beads (C20000021, Diagenode, Belgium) were added. The tubes were placed in a homogenizer (Bioruptor, Diagenode, Belgium) and sonicated for a total of 5 min (10 × 30 s on/30 s off cycle). After homogenization, 120 μL ice cold MeOH and 540 μL ice cold MTBE were added. The tubes were incubated for 1 h at 4 °C and 1000 rpm in a Thermomixer (Eppendorf, Germany). To facilitate phase separation 200 μL ice cold water was added. After centrifugation at 10,000 *g* and 4 °C for 10 min, the upper phase containing lipids were collected in fresh, cooled Eppendorf tubes. The aqueous phase was reextracted as described. The combined organic phases and aqueous phases were dried in a vacuum concentrator (SpeedVac, Thermo Fisher Scientific, USA) and stored at −80 °C until further analysis.

### RP-LC-MS/MS Analysis

The dried organic phases were reconstituted in 100 μL MeOH/*i*-PrOH (1:1, v/v). Samples were analyzed using an UHPLC system (Vanquish Flex, Thermo Fisher Scientific, Germany) equipped with a Triart C18 reversed-phase column (150 mm × 2.1 mm: 1.9 μm, 120 Å, YMC, Japan) coupled to a HESI-orbital trapping mass spectrometer (Exploris 240, Thermo Fisher Scientific, Germany). Separation was achieved by a 38 min gradient of mobile phase A (MPA) (ACN/water, 1:1, v/v) and mobile phase B (MPB, *i*-PrOH/ACN/water, 85:10:5, v/v/v) both containing 5 mM NH_4_HCO_2_ and 0.1% FA at a flow rate of 300 μL/min, a column oven temperature of 50 °C, and an injection volume of 5 μL. Gradient: 0–3 min from 10–25% MPB, 3–9 min from 25–54% MPB, 9–14 min from 54–77% MPB, 14–18 min from 77–88% MPB, 18–22 min from 88–95% MPB, 22–30 min isocratic flow, 30–30.5 min from 95–10% MPB, 30.5–38 min isocratic flow. MS measurements were carried out in data dependent top 10 acquisition mode. Survey scans were performed at a resolution of 180,000 in a scan range of *m*/*z* 150–1200. For dd-MS^2^ measurements a resolution of 15,000, minimum signal threshold of 1000, isolation window of 1.5 *m*/*z*, and stepped HCD activation energies of NCE 20, 25, 30 were set. Spectra were analyzed using Lipostar2 (v 2.1.4., Molecular Discovery, Italy). Data sets were loaded with a signal threshold of 1000. Blank subtraction, retention time filtering, and removal of lipids without MS^2^ and isotopic pattern was performed prior identification. For identification *m*/*z* tolerances of ±2.0 ppm and ±6.0 ppm for MS^1^ and MS^2^, respectively, were set. Identifications are based on the LipidMAPS database. Integration of lipid signals for identified species was performed in Skyline (version 24.1.0.199, MacCoss Lab, USA).

### BCA

After redissolving the dried aqueous phase in 150 μL water, samples were incubated for 30 min at 95 °C and 1000 rpm in a Thermomixer (Eppendorf, Germany). Every 5 min samples were taken out of the Thermomixer and ultrasonicated for 60 s. Samples were cooled down to 25 °C for 15 min. All further steps were performed as indicated for the Pierce BCA Protein Assay Kit (Thermo Fisher Scientific, Germany).

### Data Analysis

The obtained peak areas are manually type I isotopic corrected based on the assigned lipid species in Excel (see eq S1). Results from RP-LC-MS/MS and MALDI-MSI analysis and quantification were combined and further analyzed in Excel (version 2502, Microsoft, USA). Statistical analysis was performed using MetaboAnalyst 6.0.^45^ Graphs were generated in OriginPro (ver. 2022, OriginLab Corporation, USA).

## Results and Discussion

### Experimental Design and Data Analysis

The combined MALDI-MSI and RP-LC-MS/MS workflows are depicted in Figure 1. After sectioning of fresh-frozen mouse tissue, sections are separated in two batches. For each section used for quantitative spatial analysis, three adjacent sections were collected in a cooled tube for bulk analysis by RP-LC-MS/MS. The RP-LC-MS/MS data sets are used to cross-check and compare lipid identification and concentrations to MALDI-MSI results. For the development and detailed assessment of the multimodal workflow, we used mouse heart septa due to their homogeneous lipid distribution in tissue. The assessed workflow was subsequently applied to mouse kidney samples of mice fed with a normal diet (ND) or a high-fat diet supplemented with l-NAME and administration of either NO2-OA (HFD-N) or a vehicle (HFD-V).

**Figure 1:**
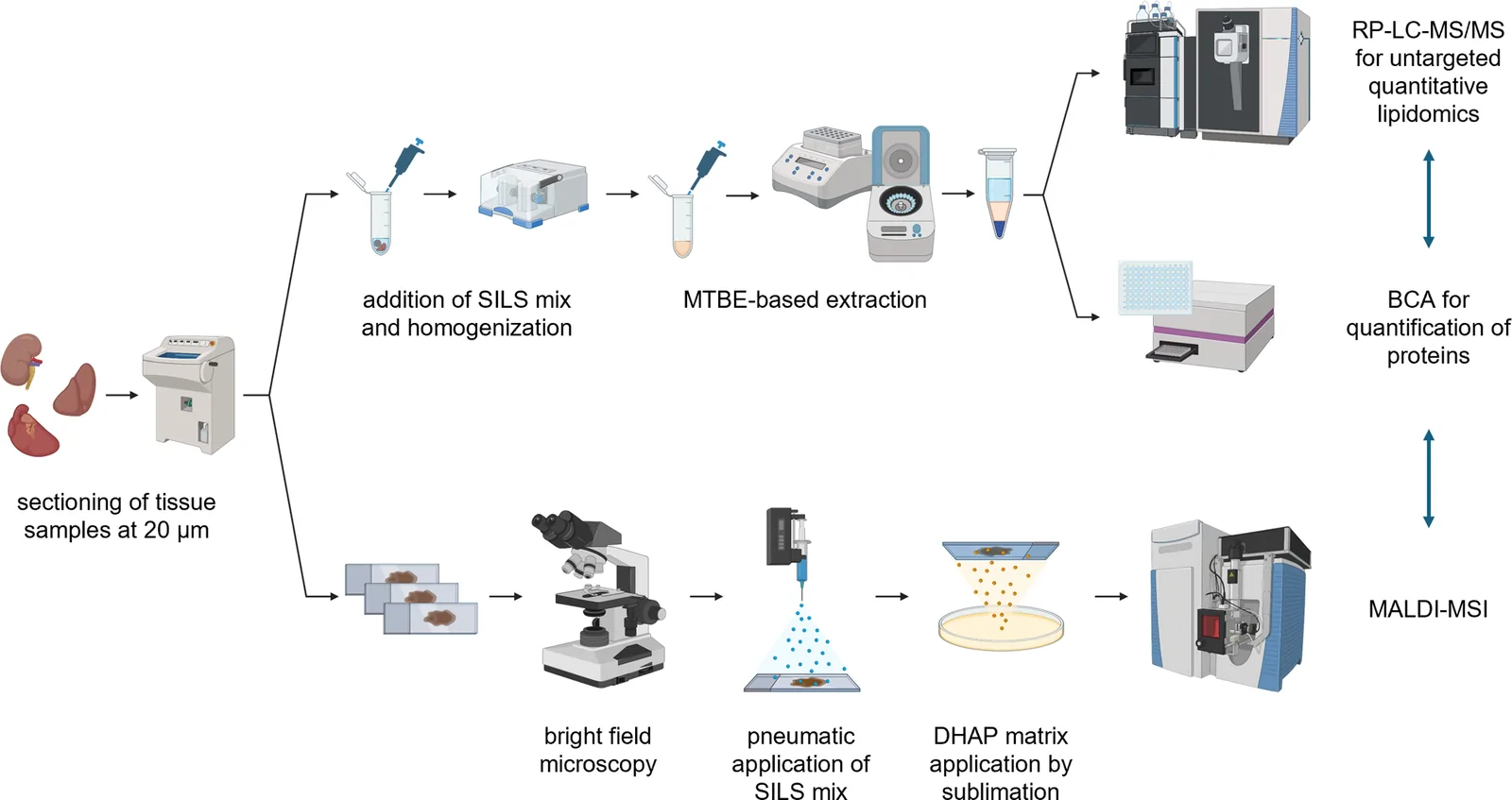
Sample preparation scheme: sample tissues were sectioned in a cryostat to obtain 20 μm thick sections. Adjacent sections were split between (i) RP-LC-MS/MS and (ii) MALDI-MSI workflow. For LC-MS/MS analysis, three sections were added in a tube with ammonium acetate solution and internal standard. After homogenization by ultrasonification with added glass beads, extraction solvents were added. Subsequent to incubation and centrifugation, the upper organic layer was dried, reconstituted, and analyzed by RP-LC-MS/MS, whereas the aqueous layer was analyzed by BCA to obtain total protein concentrations. For MALDI-MSI analysis, sections were stored vacuum sealed at −80 °C until further use. First, bright field microscopy images of each sample were obtained to ensure quality of the sections. After thawing in a desiccator for 20 min, an internal standard mix was pneumatically applied and incubated for 10 min before matrix deposition by sublimation. Samples were imaged at a resolution of 15 μm on an AP-MALDI-MSI source coupled to an orbital trapping mass spectrometer.

To generate quantitative MALDI-MSI data, a SILS mix composed of 13 lipids from individual lipid classes (see Table S1) was pneumatically applied using a home-built sprayer system with a rotating sample holder (see Figure S1). During the initial testing of our setup, it became apparent that a strict monitoring of the deposited lipid standards is necessary. Therefore, to correct for day-to-day variances in the SILS application, daily quality control of the standard spray before sample preparation was performed as described above. The signal of the applied internal standard was used to normalize signal intensities of endogenous lipid species based on the lipid class. The resulting lipid surface concentrations are compared to lipid concentrations obtained by RP-LC-MS/MS to further investigate the comparability of both methods in multimodal approaches.

Following, we will first characterize the SILS application by pneumatic spraying on glass slides and tissue sections. We have chosen consecutive mouse heart septa due to their sufficiently homogeneous structure within the analyzed area as shown in Figure S2. Although lipid distributions across biological replicates are very similar (see Figure S3), RP-LC-MS/MS analysis revealed elevated biological variability across each set of samples compared to the technical variability as shown in Figure S4. Further comparison regarding technical reproducibility and comparability of MALDI-MSI and RP-LC-MS/MS is therefore conducted on technical replicates from the same mouse heart septa of an ND mouse, to separate technical and biological variance in the method characterization. After validation of the spraying method, lipid identifications and concentration-dependent measurement errors between both methods are compared. We then characterized the technical reproducibility within our qMALDI-MSI workflow regarding lipid identification and surface concentrations before applying the characterized workflow to a set of kidney samples from ND, HFD-V, and HFD-N mice.

### Method Validation

To validate the proposed quantitative MALDI-MSI workflow, we first checked homogeneity and reproducibility of the pneumatic standard application. Therefore, the SILS mix was applied onto blank glass slides of approximately 16 × 16 mm^2^. After matrix deposition, MALDI-MSI of the whole slide was performed with a pixel resolution of 75 μm in positive-ion mode. The resulting TIC image is shown in Figure 2a. Additionally, line scans showing the signal intensity of the TIC and [PC 33:1­(d7) + H]^+^ ion through the center of the slide in *x*- and *y*-axis, as well as the outline of the size of a typical mouse heart septa are included. As indicated by the line scans, a homogeneous intensity distribution for standard lipids and thus homogeneous SILS deposition was achieved. Although intensities drop toward the edges of the glass slide, the homogeneously covered area in the center is sufficient to cover typical mouse tissue samples. Further investigation of the standard application protocol was carried out on mouse heart septa as these samples show homogeneous distributions of endogenous lipids as shown for [PC 34:1 + H]^+^ in Figure 2b. No wash out effects of endogenous lipids were observed. For reference, the outline of the tissue section is marked in white in Figure 2b. Additionally, MSI of the TIC and the internal standards [PC 33:1­(d7) + H]^+^ and [TG 48:1­(d7) + Na]^+^ are depicted. The distribution of the internal standards is homogeneous across the tissue section, with overall lower intensities compared to the off-tissue regions due to ion suppression effects. Similar results are obtained for more heterogeneous kidney tissue as shown in Figure S5. Based on these observations, we reason that a homogeneous application not only on a blank glass surface but also on tissue is achieved with the established SILS spraying protocol. However, we did experience higher signal intensities for the internal standard at the border of the tissue (roughly 30 μm width) of up to 162% for the PC standard (see Figure S6), likely resulting from the transition of the tissue to glass surface which acts as a nucleation point for the standard and matrix crystallization. Careful consideration to include these areas in quantification experiments are therefore needed.

**Figure 2:**
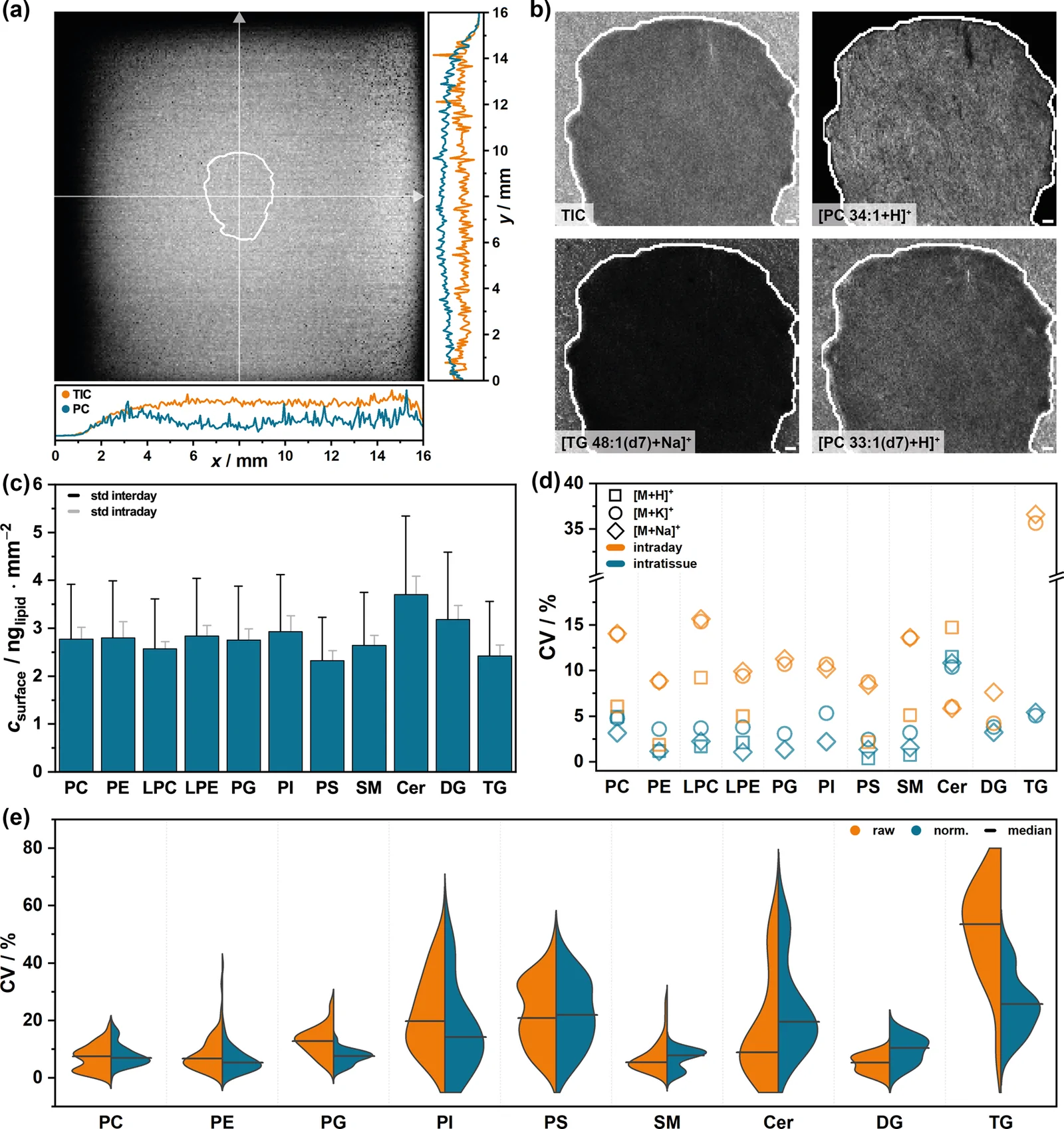
Validation of the internal standard application and normalization strategy: (a) total ion current (TIC) image of the internal standard mix pneumatically applied onto a blank glass slide and measured at 75 μm pixel resolution (total area 16 × 16 mm^2^) and line scans of the TIC (orange) and [PC 33:1­(d7) + H]^+^ (blue) in *x*- and *y*-direction through the center (indicated by white arrows) are shown. True to size outlines of a typical mouse heart septa (shown in panel (b)) are given in white. (b) MALDI-MSI at 15 μm of mouse heart septa after application of the internal standard mix and matrix. The tissue is outlined in white, scale bar 100 μm. The top row shows the TIC and endogenous PC 34:1, whereas the lower row shows selected SILS. (c) Surface concentrations of the applied internal standard determined from glass slides by RP-LC-MS/MS. Standard deviations for intraday (*n* = 3, light gray) and interday over a total of 2 weeks (*n* = 9, black) are included. (d) On-tissue CV values of internal standard application on heart tissue by MALDI-MSI for intratissue variation (blue), calculated from three regions of interest of the same measurement, and for intraday variation (orange), calculated from three separate measurements of adjacent tissue sections. (e) Intraday CV values (*n* = 3) for lipid species sorted by class determined before (raw) and after normalization representing the technical error of measurement and error of quantification, respectively.

To determine the surface concentration of the applied standard mix and its reproducibility, we applied the SILS mix to a blank glass slide daily. The slide was washed thoroughly with MeOH, the amount of internal standard quantified by RP-LC-MS/MS, and the exact area of the glass slide determined. Figure 2c shows the calculated surface concentrations for each lipid class. Error bars in black depict the standard deviation over the course of 2 weeks (*n* = 9, C*V*_min_ 39% – C*V*_max_ 47%), gray within 1 day (*n* = 3, C*V*_min_ 6% – C*V*_max_ 12%). The relatively high day-to-day variances underline the necessity of stringent daily control of the applied internal standard to ensure accurate spatial quantification.

Next, we employed the protocol to deposit SILS on the mouse heart septa. Reproducibility of the standard application on tissue was investigated intraday by comparing results of three technical replicates and intratissue by comparing results from three regions of interests of the same sample. CV values for the intensity of each internal standard of all lipid classes and the observed adducts are shown in Figure 2d. CV values were mostly below 15 and 5%, respectively, except for TGs. Additionally, an influence of the adduct type was observed: protonated species show lower CV values compared with sodium and potassium adducts. A possible explanation is the heterogeneous distribution of Na^+^ and K^+^ in the tissue compared to the constant supply of proton-donating sources throughout the tissue. The influence of the normalization strategy on the detection of endogenous lipids is shown in Figure 2e. Here, CV values for each lipid species detected in all technical replicates were calculated before (orange) and after (blue) normalization to the class-specific SILS. The former values can be viewed as the technical error of the MALDI-MSI measurements including sample handling and preparation, whereas the latter is the total error of quantification. The effect of normalization on the overall median CV is dependent on the lipid class with PCs and PSs showing no significant changes, PEs, PGs, PIs, and TGs decreasing and SMs, Cers, and DGs increasing CV values. An especially pronounced improvement of CV values by normalization was observed for TGs, which are typically difficult to analyze by MALDI-MSI. In addition to strong ion suppression effects that are known to influence TGs in MALDI-MSI, we suspect that the propensity of TG delocalization or aggregation on the polar glass substrate that mounts the tissue slides is more pronounced than that for other lipid classes. Both factors, delocalization and ion suppression, that increase the CV values for TGs can effectively be countered by normalization with homogeneously distributed SILS.

Here, we validated and characterized our SILS application workflow with regard to homogeneity and quantity of the SILS deposition by pneumatic spraying. Furthermore, we characterized the reproducibility of SILS detection on tissue and the effects of normalization on measurement errors. Before applying this workflow for the quantification of lipids, we need a means to cross-validate MALDI-MSI annotations and, consequently, identify the analytes we quantify with MALDI-MSI.

### Lipidomics Performance Characterization of MALDI-MSI and RP-LC-MS/MS

Therefore, we employed RP-LC-MS/MS to refine the annotations from MALDI-MSI and identify the lipids for which annotations and quantities could be cross-validated. An in-depth comparison of the results from both modalities regarding lipid annotations and measurement CVs for the analysis of mouse heart septum is shown in Figure 3. A proportional breakdown of lipid annotations by class from both modalities is summarized in Figure 3a. Annotations from MALDI-MSI data sets are based on accurate mass, whereas identifications from RP-LC-MS/MS are based on both exact mass and fragmentation spectra. Both identifications are based on the LipidMAPS database. In total, 577 and 468 lipids from the classes indicated in Figure 3a were identified by MALDI-MSI and RP-LC-MS/MS, respectively, with 185 lipids in common. As seen in Figure 3a, the degree of overlap between both methods depends on the lipid class. Particularly limited overlap is observed for PG, PI, and PS, which are typically measured in the negative-ion mode. However, in MALDI-MSI, these lipids form sodium and potassium adducts that are not detected or only in very low abundance under the given LC-MS conditions, making these lipids most likely only accessible in MALDI-MSI in positive-ion mode. On the other hand, we observe a larger number of oxidized lipid species as well as PAs in MALDI-MSI versus RP-LC-MS/MS, adding to the difference in detected lipids between the two data sets. Due to the different ionization methodologies, extraction of annotation lists from RP-LC-MS/MS to imply MALDI-MSI data, or *vice versa*, can be misleading and enforcing only the use of annotations present in both data sets is the best way to avoid quantification of misannotated signals. To further investigate the influence of RP-LC-MS/MS and MALDI-MSI on the obtained lipid profiles, measurement errors as a function of the calculated lipid concentrations are shown in Figure 3b,c. We expected an increase of the CV for low-abundance species, as seen in Figure 3b for the RP-LC-MS/MS data set. However, the MALDI-MSI data set in Figure 3c shows a different distribution. Here, overall elevated CV values throughout the whole dynamic range, but especially for lipid concentrations around 4 × 10^–5^ ng/mm^2^, are observed. These lipids are mainly assigned to PIs, PSs, and TGs, which are detected as sodium or potassium adducts. Therefore, we suspect a stronger influence of the local biological background and therefore ion suppression effects on the measurement error for MALDI-MSI, resulting in high CVs for abundant lipid species, whereas measurement errors in RP-LC-MS/MS are mainly influenced by the abundance of the lipids.

**Figure 3:**
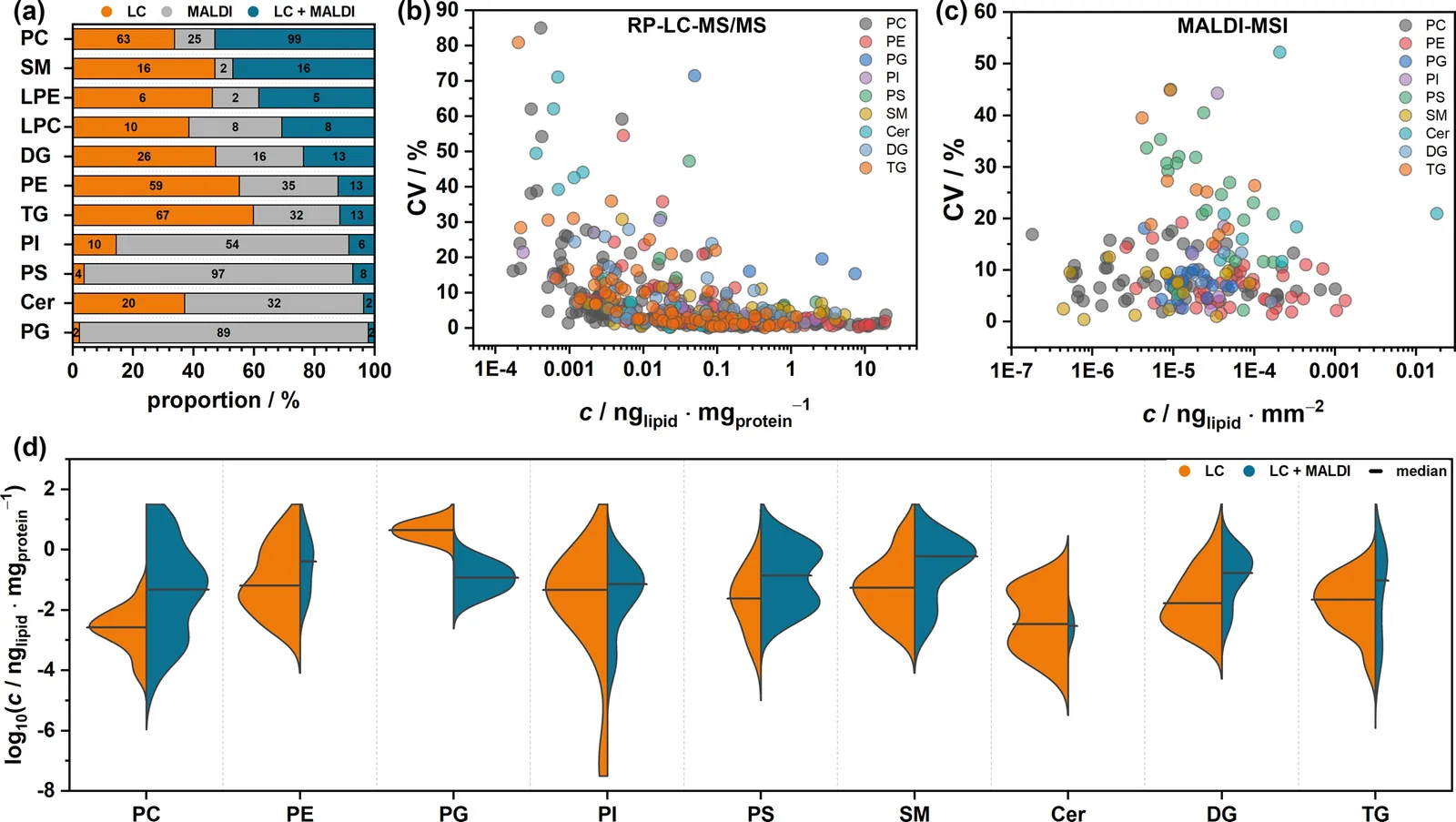
Comparison of MALDI-MSI and RP-LC-MS/MS for lipidomic analysis of heart tissue. (a) Proportional breakdown of lipid identification coverage by lipid class for MALDI-MSI and RP-LC-MS/MS from positive-ion mode measurements. Values in bars are absolute numbers of identifications. Panels (b, c) show CV values as a function of mean concentrations for RP-LC-MS/MS and MALDI-MSI measurements, respectively. Data points are color coded based on their lipid class. (d) Violin plots of lipid concentrations determined by RP-LC-MS/MS for all quantified lipid species sorted by class. Lipid species identified by only RP-LC-MS/MS or both methods are shown in orange and blue, respectively.

Next, we investigated whether lipid concentration is a descriptor for MALDI-MSI and RP-LC-MS/MS overlap. Therefore, the concentration distributions for each lipid class determined by RP-LC-MS/MS and sorted by annotation only in RP-LC-MS/MS (orange) or in MALDI-MSI and RP-LC-MS/MS (blue) are shown in Figures 3d and S7 for scatter plots. The distribution width depends on lipid class and identification method, with some showing particularly broad distributions over a wide range of concentrations such as PI. We expected that low-abundance annotations are more likely to be measured with RP-LC-MS/MS and absent in MALDI-MSI, as observed for PEs, Cers, and DGs. To our surprise, we also observe many lipids only detected by RP-LC-MS/MS with high abundances, as seen for PEs, SMs, and DGs, as well as lipids with low abundances detected by both methods, for example, in PCs and SMs. This indicates that lipid concentrations of individual compounds are not the main descriptor for an annotation in MALDI-MSI or RP-LC-MS/MS or both but that other factors such as the local, molecular makeup or incorporation into the matrix play major roles for the detection of lipids in MALDI-MSI. On the basis of these results, it is not possible to predict which individual species from the RP-LC-MS/MS data set will be likely also measured by MALDI-MSI; however, a substantial overlap can be detected for all lipid classes. For comprehensive multimodal studies, using the overlap of both annotation lists seems to be the ideal way for a qMSI workflow making use of the RP-LC-MS/MS for quality control and minimizing the number of erroneous annotations and quantification results.

### Annotation Number and qMALDI-MSI of WT Mouse Heart Septa

Next, we investigated the technical reproducibility of our qMALDI-MSI workflow on three adjacent mouse heart septa sections from ND mice. The number of repeated identifications across three replicates is given in Figure 4a. The number of lipids identified in all measurements or in two of three measurements, i.e., the reproducibility of the annotations, varies with lipid class. For example, polar lipids with permanent positive charges such as SM, LPC, and PC are more likely to be measured in all replicates compared to neutral lipids such as DG and TG or lipids with permanent negative charges such as PI. Lipid classes with a lower annotation reproducibility over three replicates are typically the lipid classes exhibiting lower intensities in MALDI-MSI (see Figure S8) and form Na^+^ and K^+^ adducts. As shown in Figure 2d, these adducts are associated with elevated fluctuations in signal intensities compared to protonated ions likely contributing to high variances in the annotation count. Figure 4b–f shows surface concentrations of lipids detected in each technical replicate. Concentrations range from less than 0.05 to 10 ng/mm^2^ depending on lipid class and species. To the best of our knowledge, there are no comparable studies to spatially quantify lipid species in mouse heart tissue. However, Vandenbosch et al. reported surface concentrations for different mouse brain regions determined by a similar workflow.^38^ In comparison, our calculated concentrations are roughly one to two orders of magnitude smaller, which can be the result of organ specific differences of lipid composition or quality control checks of applied SILS amounts. Comprehensive lipidomics analysis across different rodent tissues by LC-MS revealed unique lipid compositions for each tissue type, including significant differences down to lipid species level for mouse brain and heart tissue.^46–48^ We further cross checked our data by comparing our MALDI-MSI results with ratios of quantified lipids by differential ion mobility MS/MS data published by Cao et al. in WT mice heart.^49^ For example, the ratio of PC 36:4 to PC 34:1 is 1.8 and 1.7 in Cao et al. and our data, respectively. But also for the ratio of other lipids good agreement between our and the data from Cao et al. is achieved with better agreement for lipids of high abundance. Overall measurement errors of our data follow a similar trend as observed for the repeatability of lipid identifications in Figure 4a with the highest CV of 26% observed for TGs and lowest of 7% for SM.

**Figure 4:**
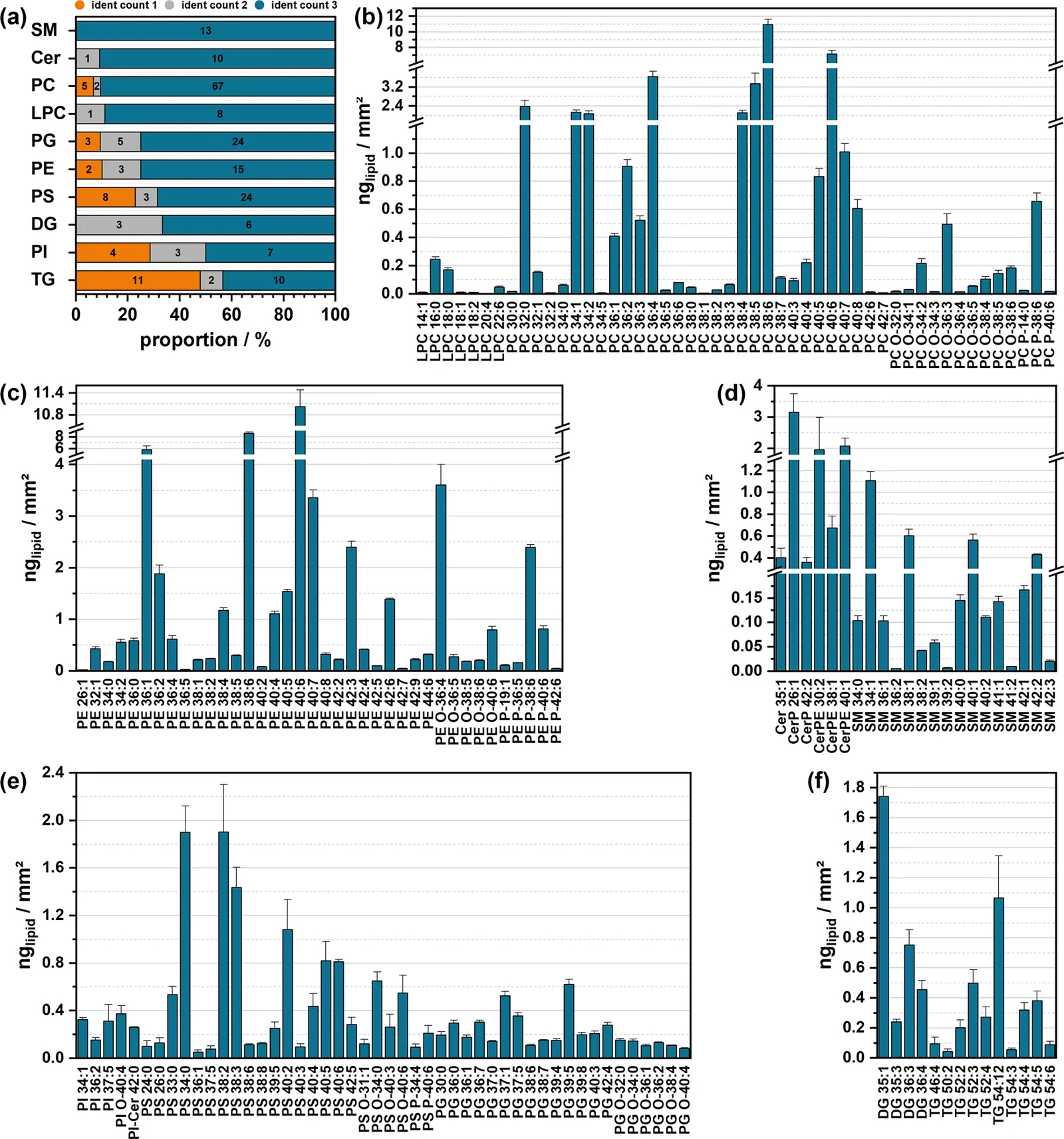
Technical reproducibility for qMALDI-MSI of lipids in three adjacent mouse heart septa sections from mice fed with a normal diet (ND) by MALDI-MSI. (a) Proportional breakdown of lipid annotation counts by lipid class in three replicates. (b–f) Surface concentrations of quantified lipids sorted by class measured in all three replicates.

### Quantitative Analysis of ND, HFD-V, and HFD-N Kidney by MALDI-MSI and RP-LC-MS/MS

After thoroughly characterizing our qMALDI-MSI method and benchmarking against RP-LC-MS/MS regarding measurement, quantification, and reproducibility of annotations on mouse heart septa, we applied the method to mouse kidneys to study the lipidomic alteration of the kidney lipidome upon different diets and treatments. Three biological replicates of three mouse cohorts (ND, HFD-V, and HFD-N) were measured according to the workflow in Figure 1. First, we investigated the influence of different normalization strategies on the obtained MS images, as shown in Figure 5a. The top row shows raw images without normalization of the different adducts of the endogenous, omnipresent lipid PC 34:1, the middle row shows the same images after TIC normalization, whereas the lower row shows normalization to the respective adduct of the internal PC standard PC 33:1­(d7). In this case, TIC normalization does not influence the perceived ion distribution compared with MSI results without normalization. In both cases, a high contrast between the different tubule structures in the cortical labyrinth of the kidney is observed, with certain areas showing almost no signal at all. In comparison, the images obtained after normalization to the SILS showed a significantly increased signal abundance in these areas. As described earlier, the use of an internal standard does not reduce the effects of ion suppression; however, it can help in visualizing and mitigating those influences on the presented results as demonstrated here at high spatial resolution.

**Figure 5:**
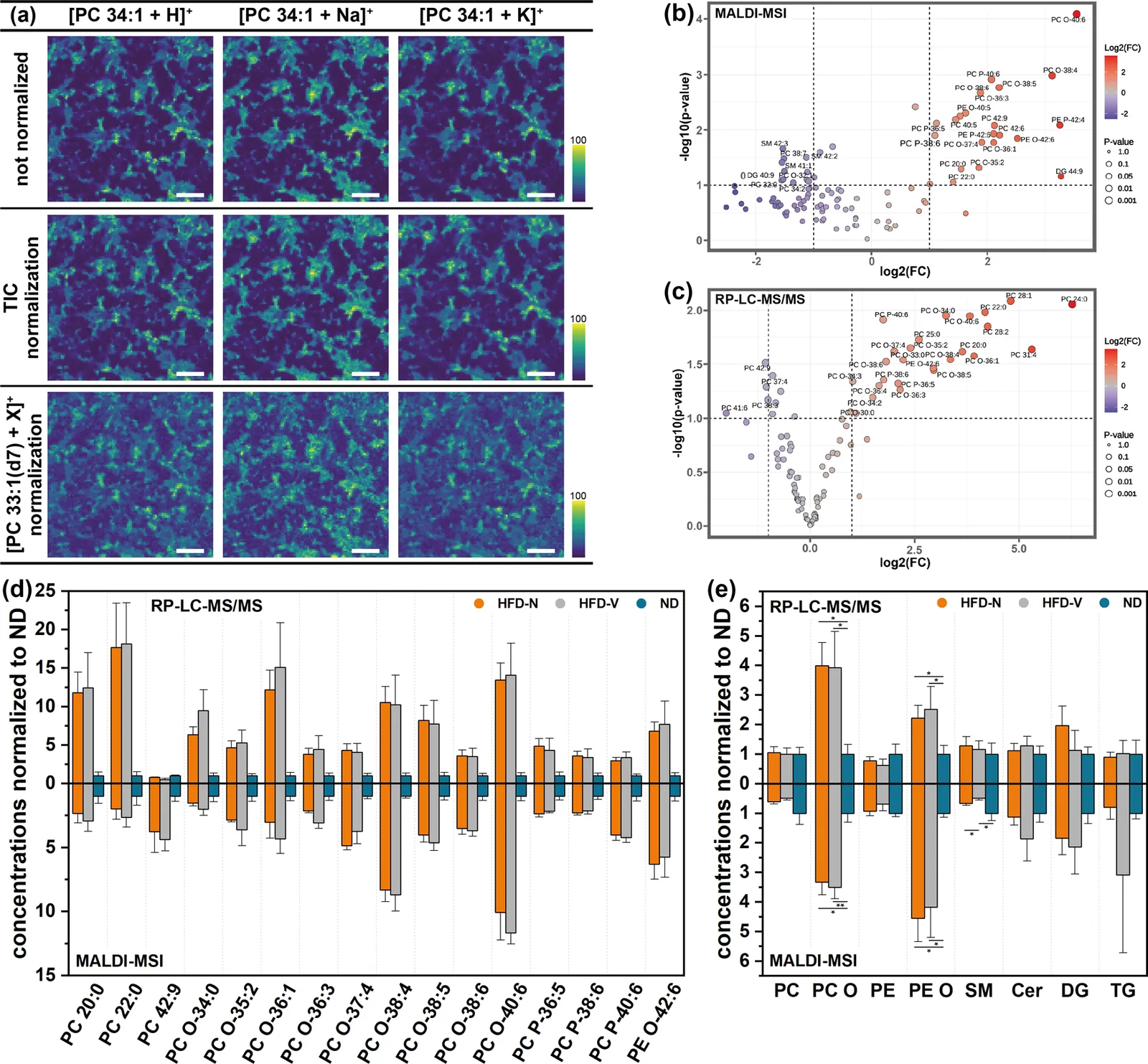
Comparison of quantitative MALDI-MSI and RP-LC-MS/MS based lipidomic analysis of mouse kidney tissue from ND, HFD-V and HFD-N mice. (a) Influence of different normalization techniques on the obtained and perceived MALDI-MSI results, scale bar 250 μm. Quantified lipid profiles from HFD-V vs ND based on MALDI-MSI and RP-LC-MS/MS data are compared in volcano plots (b, c), respectively. Only lipids that have been detected by both methods and in each biological replicate have been used for the analysis. (d) Bar graph comparing lipid abundances normalized to ND for highly regulated (FC > 2, *p* < 0.05) lipid species determined by both MALDI-MSI and RP-LC-MS/MS. (e) Sum concentration for quantified lipid classes obtained by MALDI-MSI and RP-LC-MS/MS normalized to ND in positive-ion mode. Only statistically significant differences are indicated: p*<0.05, **<0.01.

Lipidomics data sets from MALDI-MSI and RP-LC-MS/MS of mouse kidneys were filtered to only contain lipid species detected in all three replicates by both modalities. To investigate possible changes on the lipidome level due to dietary changes in more detail, volcano plots comparing ND and HFD-V based on quantitative MALDI-MSI and RP-LC-MS/MS data are presented in Figure 5b,c, respectively. Multiple lipids, especially PC and PC-O/PC-P, are in both modalities highly upregulated. The overlap of these highly upregulated lipid species in the HFD-V mouse kidneys is shown in Figure 5d normalized to the ND concentrations. Most of these lipids show very similar trends across both platforms, such as PC O-34:0, PC O-36:1, PC O-40:6, and PC P-38:6 with a significant concentration increase in both HFD groups compared to the ND. However, significant changes between HFD-V and HFD-N were not observed. For example, PC O-34:0 has a fold change of 9.5 based on LC-MS and 2.0 based on MALDI-MSI for HFD-V compared with ND. Similarly, PC O-36:1 and PC O-40:6 have fold changes between HFD-V and ND of 15.1/4.3 and 14.1/11.6 in LC-MS/MALDI-MSI, respectively. Our data imply that the overall trend in concentration changes of significantly regulated lipids can be observed by both modalities if strict quality control measures are applied, however, the determined magnitude of these changes can differ. On average, LC-MS-based fold changes for HFD-V for the lipids shown in Figure 5d are 2.1 times higher compared to MALDI-MSI-based results.This observation can be explained by the lower dynamic range typically observed in MALDI-MSI compared to LC-MS measurements (Figure 3b,c). To further investigate the comparability of both methods, Pearson correlation plots of lipid concentrations normalized to the sum concentration are shown in Figure S10, with Pearson correlations ranging from 0.80 (HFD-N) to 0.89 (ND). This correlation analysis reveals that for the majority of data points, they exhibit higher concentrations in LC-MS compared to MALDI-MSI for abundant lipid species. But also a small number of lipids exist in which mainly PEs and PCs show high abundance in MALDI-MSI but very low abundance in LC-MS. Despite these general trends, a correlation between 0.80 and 0.89 reveals that for the large majority of filtered lipids, good correlation of the quantification values between the two mass spectrometric analysis pipelines exists.

The increased concentration of ether phospholipids for HFD compared to ND mice are not only observed on the level of individual lipids but also in the class wise comparison in Figure 5e with a fold change of 3.9 and 3.5 for LC-MS and MALDI-MSI, respectively, between HFD-V and ND. Besides regulating membrane fluidity and intracellular signaling, ether lipids and especially plasmalogens are known to be potent endogenous antioxidants.^50–52^ Pietiläinen et al. have shown a negative correlation between obesity and circulating ether lipid concentrations in humans.^53^ This reduction might lead to an increase in oxidative and ER stress, disruption of cellular membranes, and inflammation, contributing to the disease progression.^50^ However, Schweizer et al. reported increased plasmalogen levels in white adipose tissue (WAT), the primary lipid storage tissue, in obese mice.^54^ As reactive oxygen species (ROS) levels are elevated in WAT of obese mice,^55^ increasing plasmalogen concentrations in white adipocytes has been reported to have a protective function. Pietiläinen et al. observed similar trends in subcutaneous WAT (SAT) in humans.^56^ The increased ether lipid concentrations in HFD-mice compared to the ND group are in line with the reported observation for WAT. The lipidome is adapting to an increase of ROS production by elevating endogenous antioxidants in the kidney.

## Conclusions

In this study, a workflow that integrates quantitative MALDI-MSI and RP-LC-MS/MS of lipids was presented and thoroughly investigated with regard to SILS application, intraday as well as interday reproducibility, and quantifiable lipid annotations. Our workflow enables spatial quantification based on a pneumatically applied SILS with strict control of SILS surface concentrations and lipid annotations by RP-LC-MS/MS.

Our results suggest that frequent control of the internal standard application is necessary to ensure accurate quantification results in MALDI-MSI. This is due to the fact that losses of SILS during deposition and inter/intraday variabilities can lead to pronounced systematic changes of quantification results if quality control checks are not embedded in the workflow. Additionally, our study brings attention to thoroughly controlling lipid annotations via RP-LC-MS/MS before MALDI-MSI quantification. This is because signal intensities in MALDI-MSI and associated CVs do not follow a trend that can be inferred from RP-LC-MS/MS, requiring the control of MALDI-MSI annotations and quantitation by RP-LC-MS/MS as demonstrated here. We then applied the established workflow to a two-hit HFpEF mouse model to characterize lipidomic changes in the kidney. When qMALDI-MSI is quality controlled as established here, the relative trend of RP-LC-MS/MS quantitation results is reproduced by MALDI-MSI. In particular, our data suggest an increase in ether phospholipids, which are known to be endogenous antioxidants. These findings are supported by multiple studies reporting an increase in tissues such as WAT in obese mice and humans as a protective measure against oxidative stress.^54,56^

This work is in line with recent developments and efforts to achieve spatial quantitation of lipids in tissue, which will greatly contribute to the understanding of disease mechanisms with highly localized tissue alterations, as seen, e.g., in the tumor microenvironment. Still limitations of the workflow exist that need to be addressed in the future: (a) MALDI-MSI quantitation results are based on a single point calibration which does not allow to characterize linearity of detector response as well as tissue-specific matrix effects; (b) use of one internal standard per lipid class does not account for differences in ionization efficiencies within lipid classes due to variations in acyl chain length and degree of unsaturation; (c) further explore dual polarity MALDI-MSI to ease the analysis of certain lipid classes, such as PE, PI, PS, and PG; (d) characterize the influence of different matrices on quantitative lipidome coverage; (e) determine LOD and LOQ for quantitative MSI for different analytes; (f) other quantitative lipidomics platforms and ionization techniques, such as shotgun MS and DESI-MSI, could improve the throughput of the workflow; (g) the inability to define mol/m^3^ in qMSI without introducing too many assumptions requires to compare relative qMSI and RP-LC-MS/MS results; (h) automation of the pneumatic standard application to reduce interday variances and strengthen the workflow’s robustness; and (i) an automated software pipeline to seamlessly integrate qMSI and RP-LC-MS/MS results is not available. With these limitations in mind, we hope that our in-depth comparison of MALDI-MSI and RP-LC-MS/MS regarding lipid coverage and measurement errors set ground for future work in quantitative lipidomics building on the herein described workflow that combines qMSI with necessary quality control measures enabling to capture spatial molecular alteration on a quantitative level.

## Supplementary Material



## Notes

Additional equations, tables, and figures, including equation to calculate isotopic correction type 1, composition of the SILS mix spray solution, picture and schematic of the spraying setup, MALDI-MSI of consecutive mouse heart septa sections and of nine biological replicates to assess tissue homogeneity, comparison of CV values for lipids in heart septa determined by MALDI-MSI and RP-LC-MS/MS, additional MALDI-MSI of SILS application on heart and kidney tissue in comparison to endogenous lipids, axial intensity distribution of SILS on tissue, scatter plots of lipid concentrations by RP-LC-MS/MS of mouse heart septa sorted by lipid class, scatter plots of lipid intensities and surface concentrations by MALDI-MSI of mouse heart septa sorted by lipid class, sum concentrations of lipid classes by RP-LC-MS/MS and MALDI-MSI and lipid composition of mouse kidney, Pearson correlation of lipid concentrations by RP-LC-MS/MS and MALDI-MSI of lipids quantified in mouse kidney (PDF)
