# Supplementary material for: Quantitative MALDI-MSI Workflow for Lipids with a LC-MS-Based Quality Control Pipeline
Source: ACS Meas Sci Au. 2026 Jun 6;6(4):1194–205. doi: 10.1021/acsmeasuresciau.6c00083 (PMC13495717; doi:10.1021/acsmeasuresciau.6c00083)
Supplement: Supplementary file 1 [file tg6c00083_si_001.pdf]

Quantitative MALDI-MSI workflow for lipids with a LC-MS based quality control pipeline

Felix-Levin Hormann[a] and Sven Heiles[a, b] *

[a] Leibniz-Institut für Analytische Wissenschaften - ISAS - e.V., Otto-Hahn-Straße 6b, 44139 Dortmund, Germany

[b] Lipidomics, Faculty of Chemistry, University of Duisburg-Essen, Universitätsstrasse 5, 45141 Essen, Germany

Table of Contents

Equation S1	Equation to calculate isotopic correction type I	S2
Table S1	Composition SILS mix spray solution	S2
Figure S1	Picture and schematic of the pneumatic spray setup	S2
Figure S2	MALDI-MSI of endogenous lipids in consecutive mouse heart septa sections	S3
Figure S3	MALDI-MSI of PC 38:6 in biological replicates of mouse heart septa	S3
Figure S4	CVs for endogenous PCs quantified by RP-LC-MS/MS and MALDI-MSI	S4
Figure S5	MALDI-MSI of SILS on mouse heart septa and kidney	S4
Figure S6	Intensity distribution of SILS and PC34:1 on tissue by MALDI-MSI	S5
Figure S7	Scatter plots of lipid concentrations by RP-LC-MS/MS by lipid class	S5
Figure S8	Scatter plots of lipid intensities and concentrations by MALDI-MSI by lipid class	S6
Figure S9	Lipid concentrations determined in mouse kidney samples	S6
Figure S10	Pearson correlation of lipid concentrations by RP-LC-MS/MS and MALDI-MSI	S7

*Address correspondence to Prof. Dr. Sven Heiles:

Leibniz-Institut für Analytische Wissenschaften - ISAS - e.V.

Otto-Hahn-Straße 6b

44139 Dortmund, Germany

Phone: +49 231 13 92 4202

e-mail: sven.heiles@isas.de

$$I_{total} = I_0 \cdot \left(1 + 0.0109n + \frac{0.0109^2 n(n-1)}{2}\right)$$

Equation S1. Equation to calculate isotopic correction type 1

Table S1. Composition of the stable isotope labelled internal standard mix spray solution in methanol/water (1:1, v/v).

name	chemical formula	molecular mass	*c* / nmol·mL^{-1}	*β* / µg·mL^{-1}
PC 15:0-18:1(d7)	C41H73D7NO8P	753.11	44.26	33.3
LPC 18:1(d7)	C26H45D7NO7P	528.72	63.05	33.3
PE 15:0-18:1(d7)	C38H67D7NO8P	711.03	46.88	33.3
LPE 18:1(d7)	C23H39D7NO7P	486.64	68.50	33.3
PG 15:0-18:1(d7)	C39H68D7O10P	764.02	43.63	33.3
PI 15:0-18:1(d7)	C42H72D7O13P	847.13	39.35	33.3
PS 15:0-18:1(d7)	C39H67D7NO10P	777.02	42.90	33.3
TG 15:0-18:1(d7)	C51H89D7O6	812.37	41.03	33.3
DG 15:0-18:1(d7)	C36H61D7O5	587.98	56.69	33.3
MG 18:1(d7)	C21H33D7O4	363.59	91.68	33.3
CE 18:1-d7	C45H71D7O2	658.16	50.65	33.3
SM(d18:1/18:1)-d9	C41H72D9N2O6P	738.12	45.16	33.3
Cer(d18:1(d7)/14:0)	C33H58D7NO3	530.92	62.78	33.3

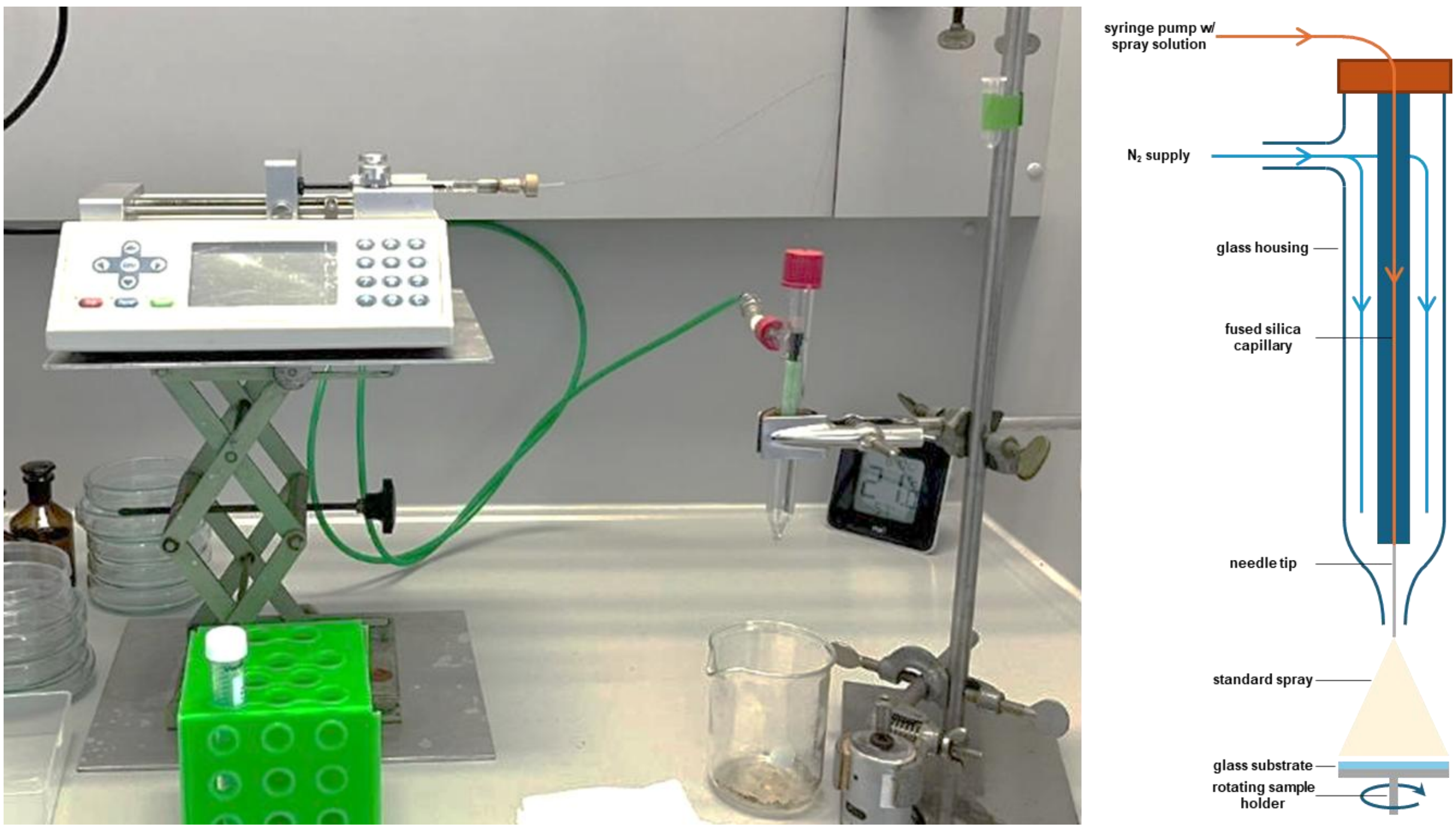

Figure S1. Picture and schematic representation of the spraying setup for pneumatic SILS application.

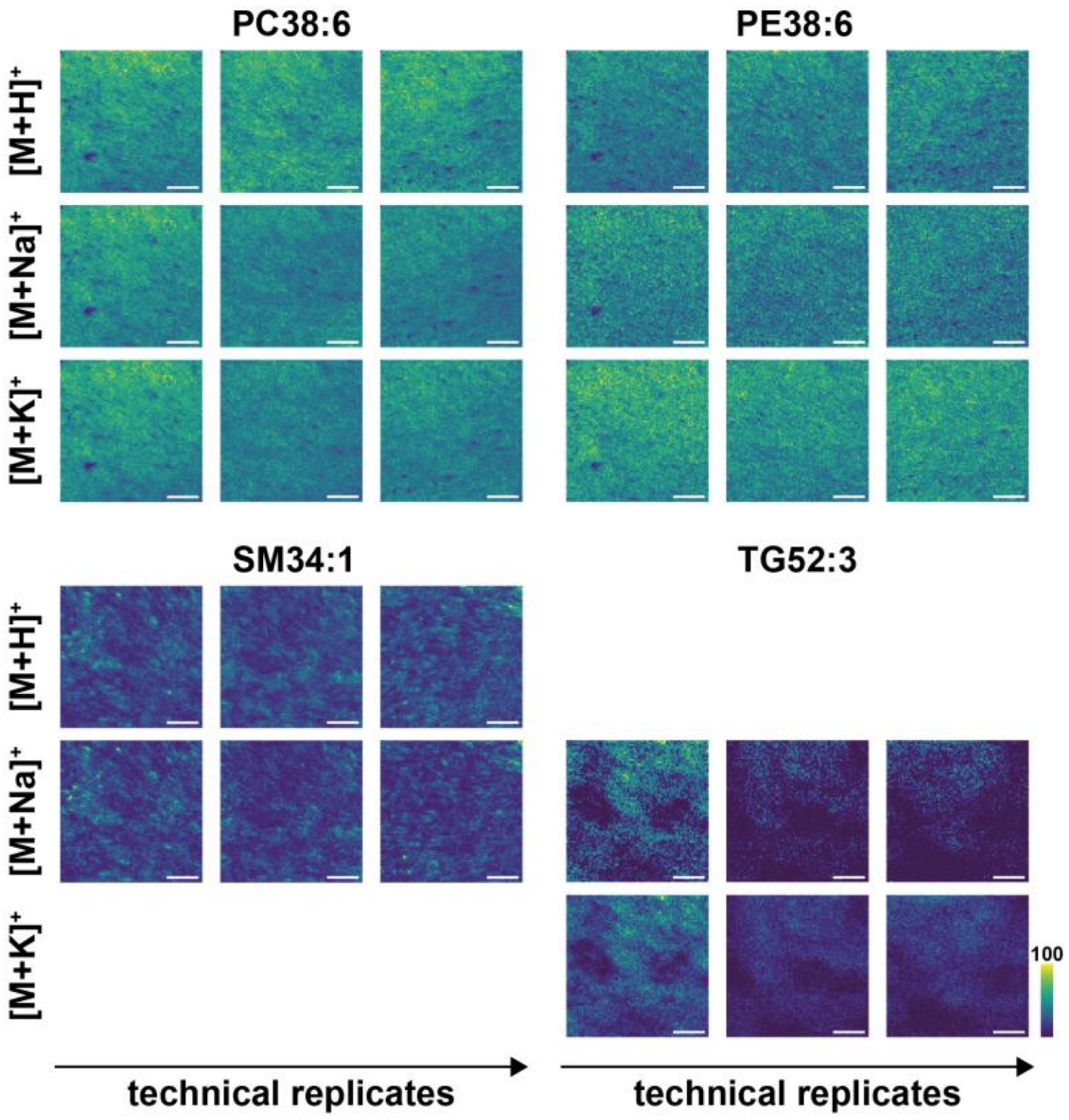

Figure S2. MALDI-MSI of endogenous lipids in three consecutive ND mouse heart septa sections (20 µm thickness). Although measurement areas within the set of sections were not aligned, strong similarities between the obtained ion distributions are observed. SMs and TGs show distinct ion distributions, that are qualitatively reproduced within the set of replicates. Measurement area: 75x75 px at 15 µm pixel size, scale bar 250 µm.

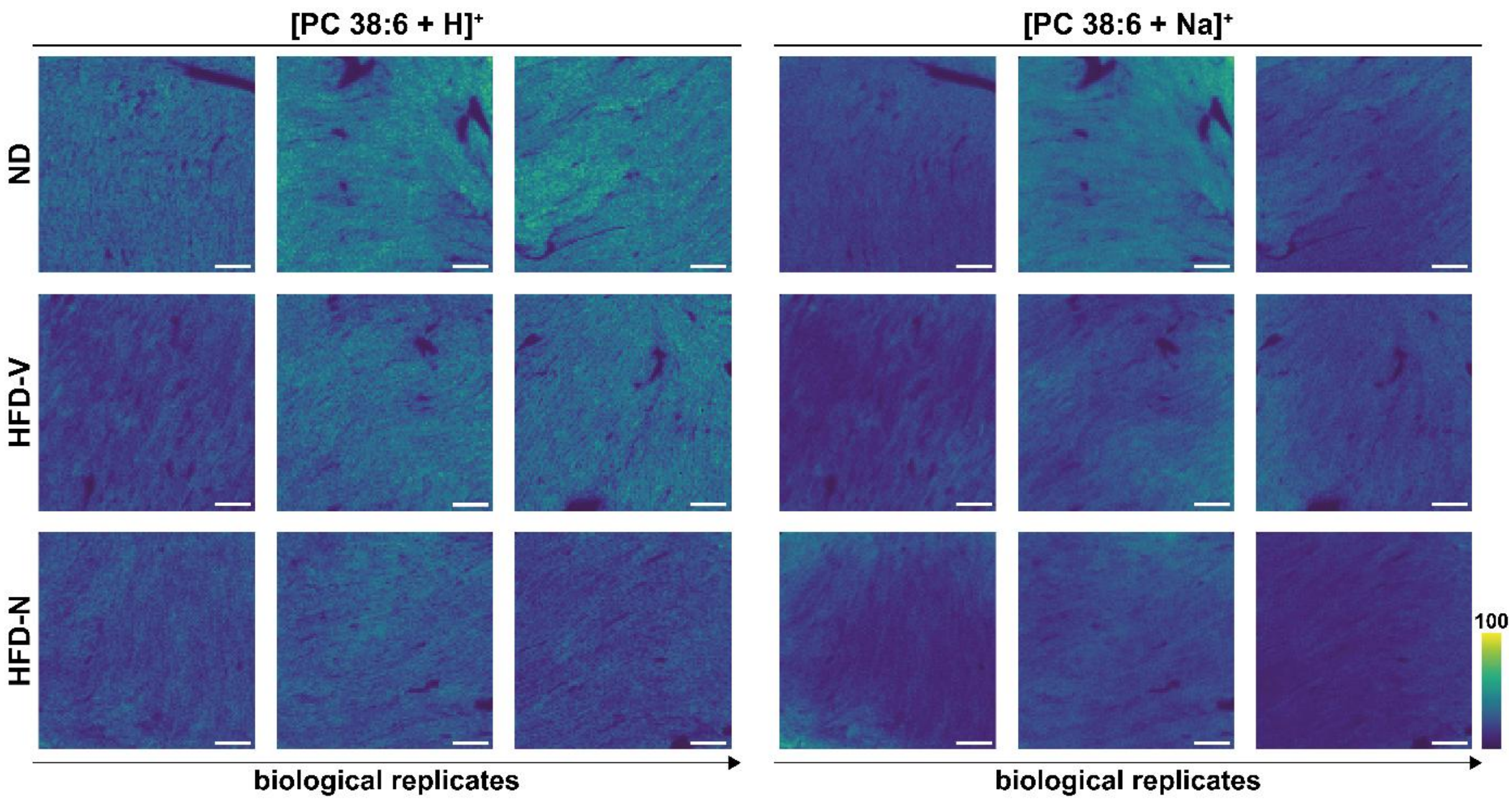

Figure S3. MALDI-MSI of endogenous PC 38:6 in mouse heart septa. A total of nine biological replicates with three replicates per group are shown. Although measurement areas within the set of sections were not aligned, similarities between the obtained ion distributions are observed, albeit varying signal intensities. Measurement area: 100x100 px at 15 µm pixel size, scale bar 250 µm.

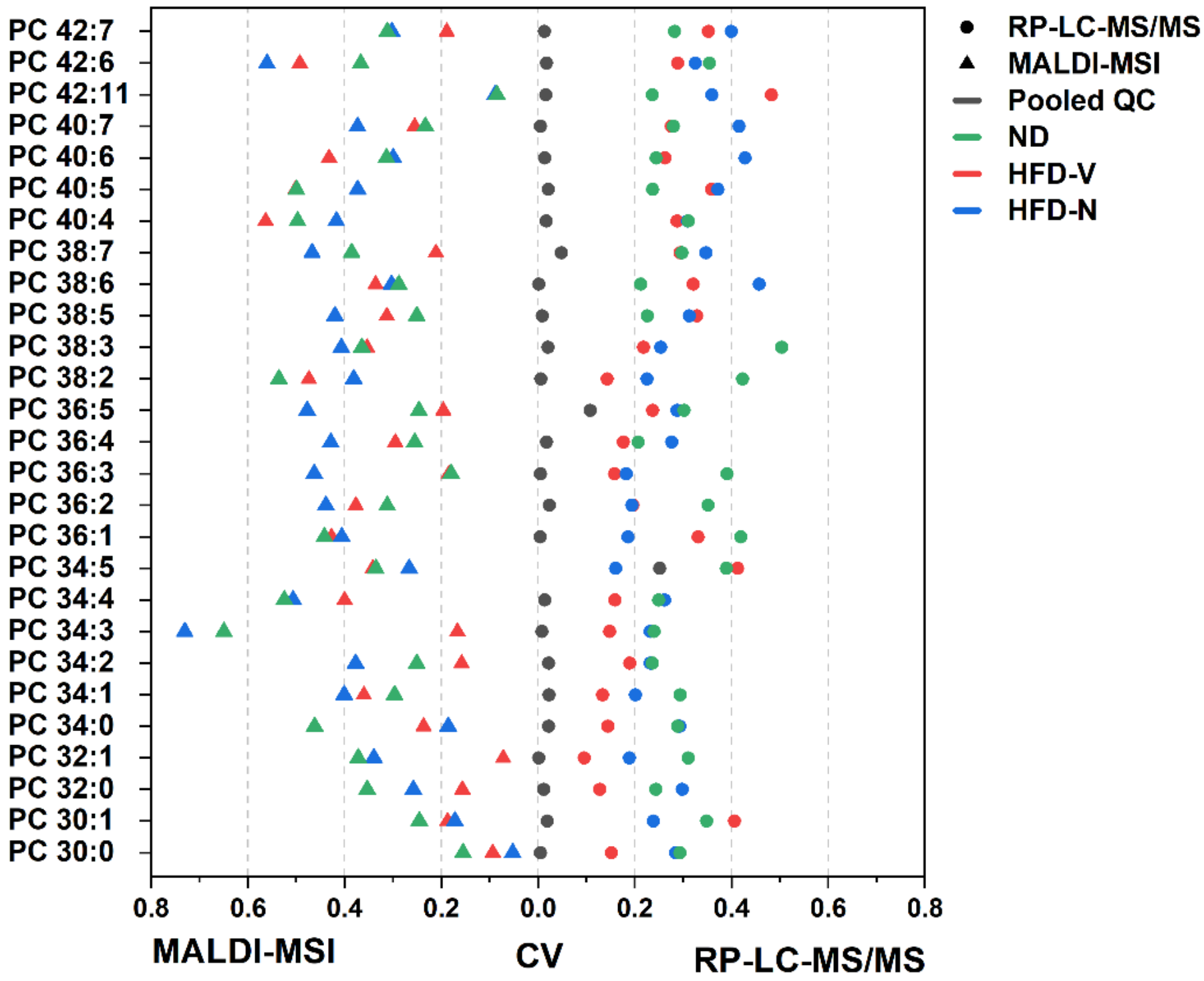

Figure S4. Comparison of CV values for endogenous PCs quantified in three biological replicates per group by MALDI-MSI (triangles) and RP-LC-MS/MS (circles). Additionally, grey circles represent CVs determined from three measurements of a pooled QC sample (containing all nine biological replicates) by RP-LC-MS/MS. The latter representing the overall technical error of RP-LC-MS/MS measurements under the given conditions. As evident, CVs for each group are significantly higher than the determined technical error, leading to the conclusion of high biological variance within each group. CV values of MALDI-MSI analysis mirror the results from RP-LC-MS/MS.

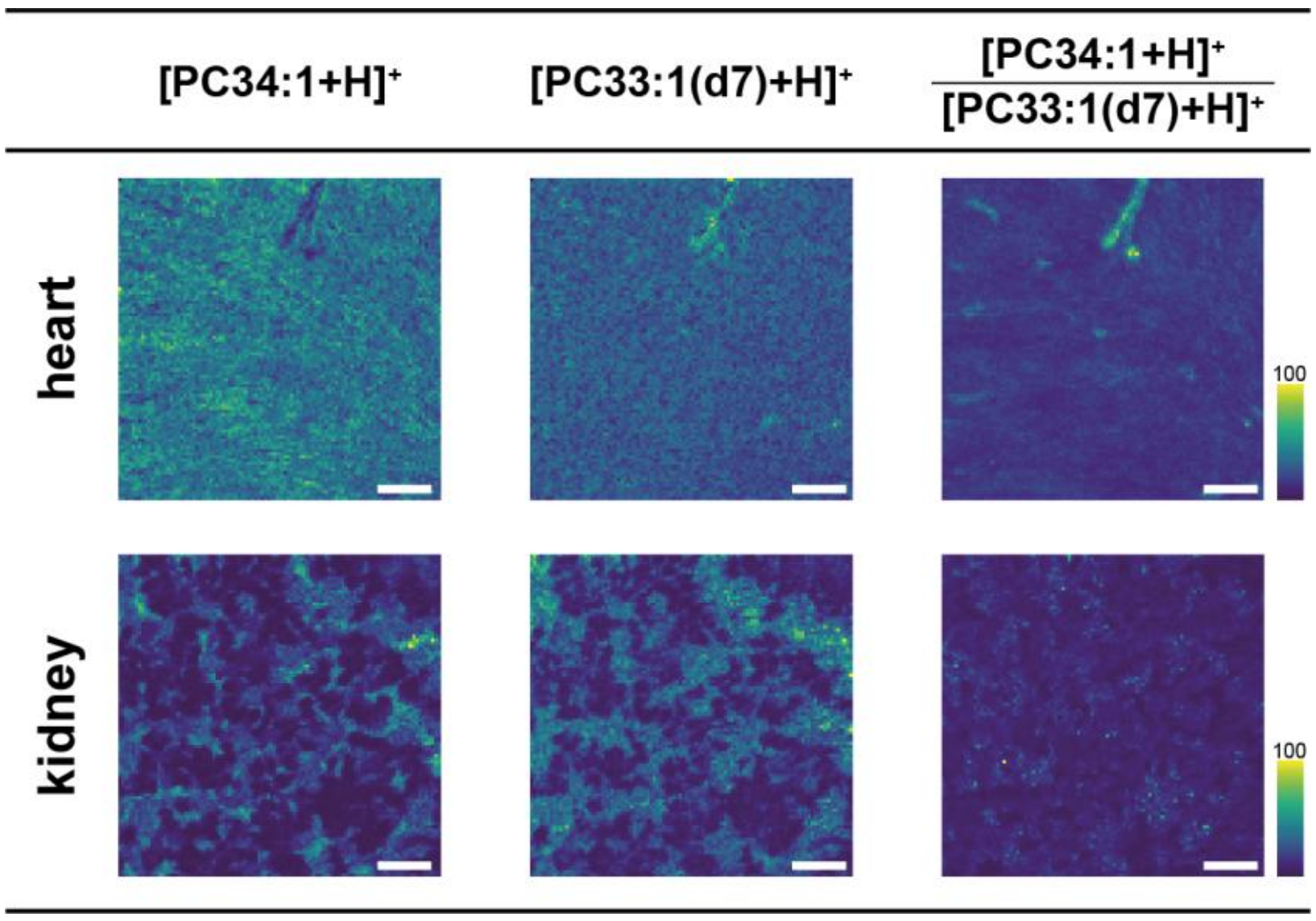

Figure S5: Comparison of SILS application on heart septa (top row) and kidney (bottom row). Depicted are MALDI-MSI of the endogenous lipid PC 34:1, the SILS PC 33:1(d7), and the ratio of PC 34:1 to PC 33:1(d7) (columns from left to right). The ion intensity distribution of the SILS follows a similar trend when compared to an endogenous lipid as seen from the MS images depicting the ratio of the lipids on the right. The observed hotspots in the upper region of the heart ratio image are due to blood vessels which correspond to very low or no signal of the endogenous lipid (compare left column). Measurement area: 75x75 px at 15 µm pixel size, scale bar 250 µm.

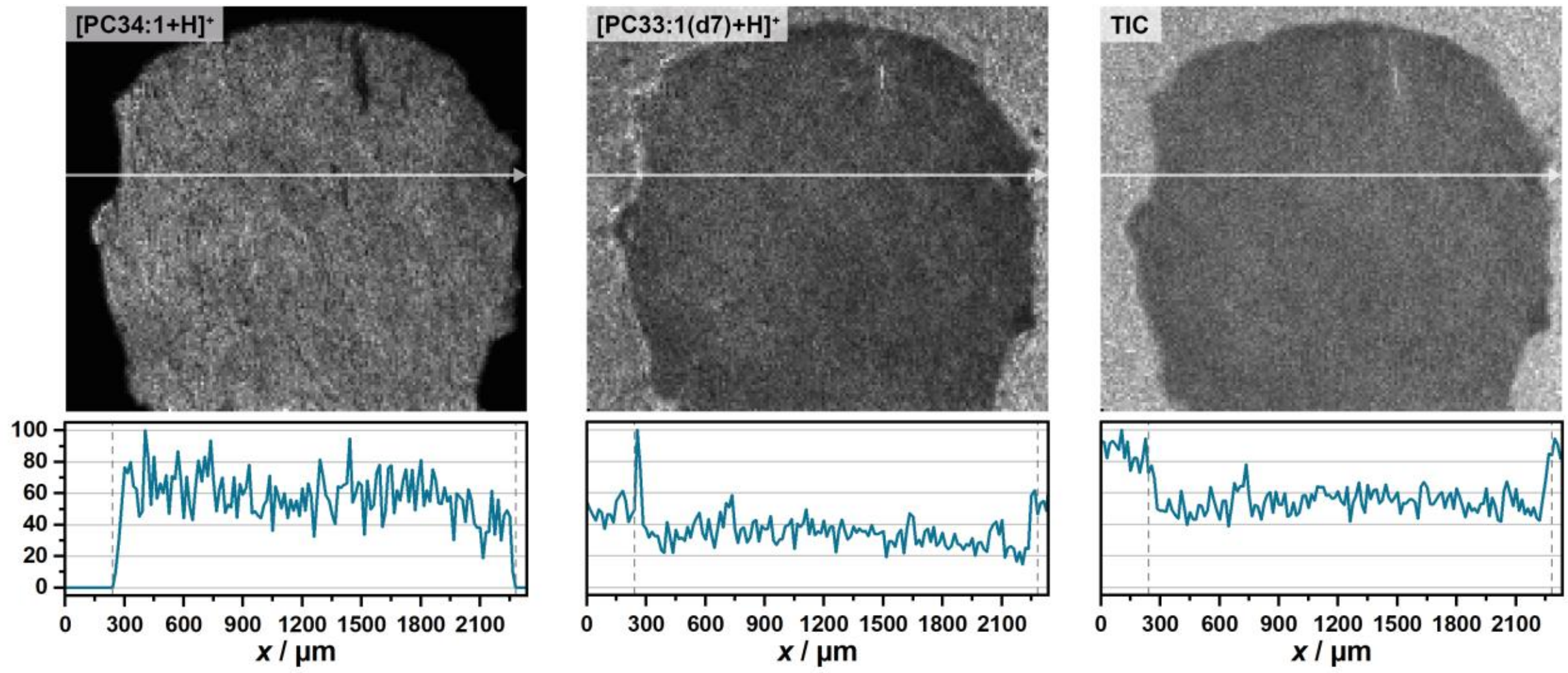

Figure S6. MALDI-MSI at 15 µm of mouse heart septa after application of the internal standard mix and matrix. The endogenous lipid [PC 34:1 + H]$^+$, the internal standard [PC 33:1(d7) + H]$^+$, and the TIC with corresponding line scans of the normalized intensity along the indicated axis shown below are shown. The edges of the tissue are indicated by grey dashed lines determined by the signal drop of the endogenous lipid. An increase of the standard signal is observed by an increase of intensity around the edge, especially on the left side.

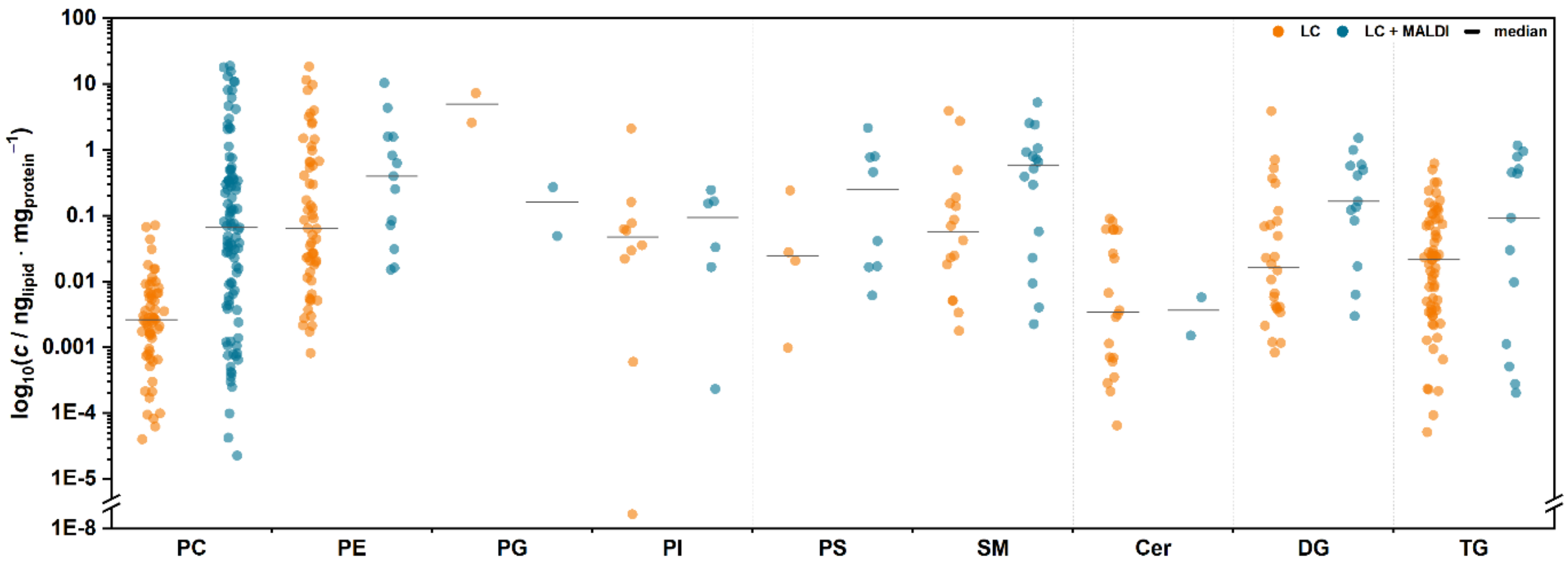

Figure S7. Scatter plots of lipid concentrations determined by RP-LC-MS/MS for all quantified lipid species sorted by class. Lipids identified by only RP-LC-MS/MS or both methods are shown in orange and blue, respectively. The median value is indicated by a black solid line.

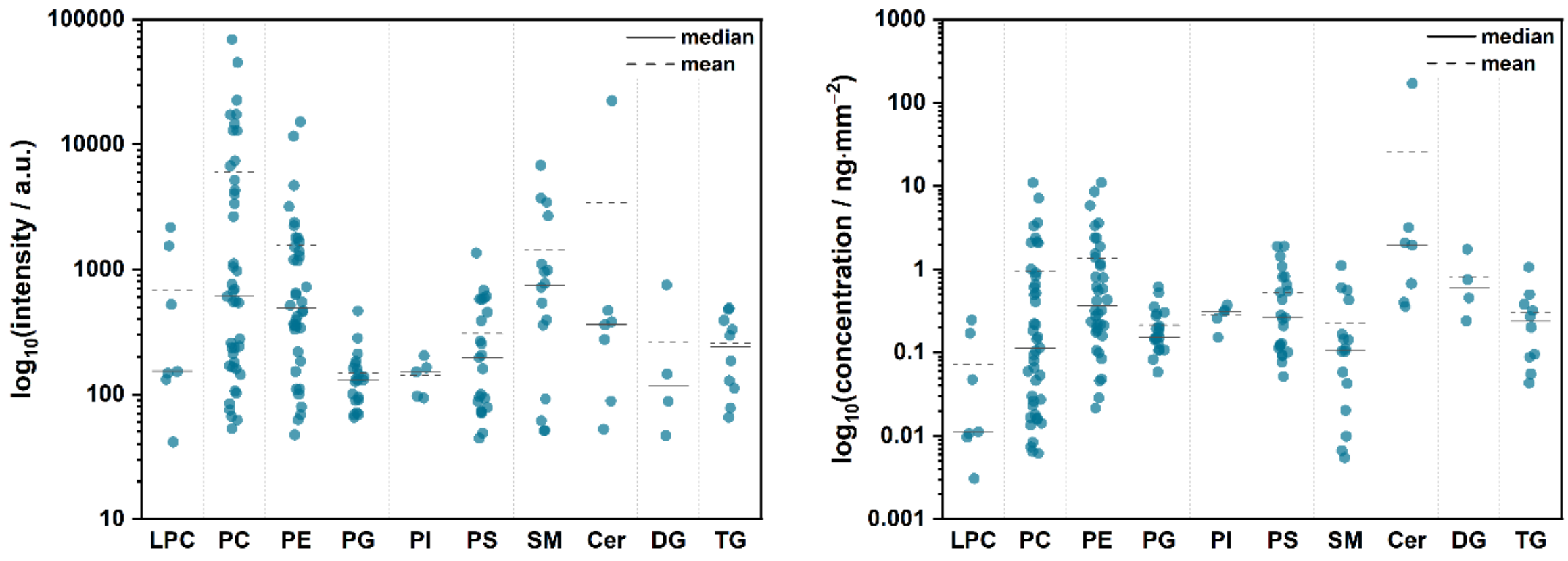

Figure S8. Data based on MALDI-MSI of three technical replicates of mouse heart septa. Left: Mean raw intensity values of lipid species sorted by lipid class. Right: Mean surface concentrations of lipid species sorted by lipid class. Median and mean of all lipid species per class are given by solid and dashed line, respectively.

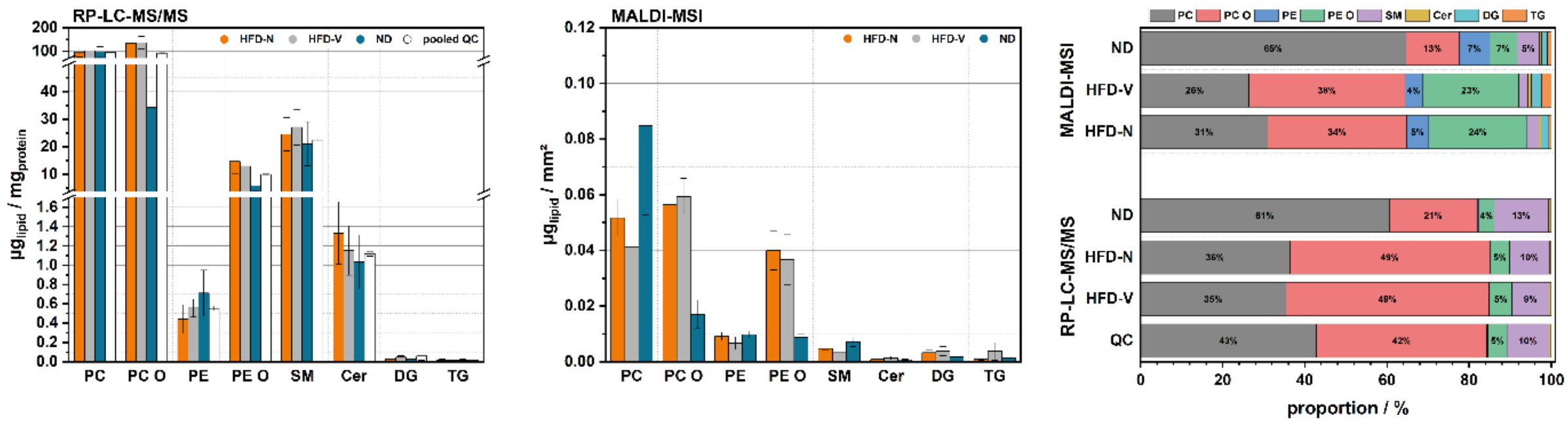

Figure S9. Sum concentration by lipid class for RP-LC-MS/MS (left) and MALDI-MSI (middle) of mouse kidney. Proportional breakdown of lipid composition for mouse kidneys of HFD-N, HFD-V, and ND mice.

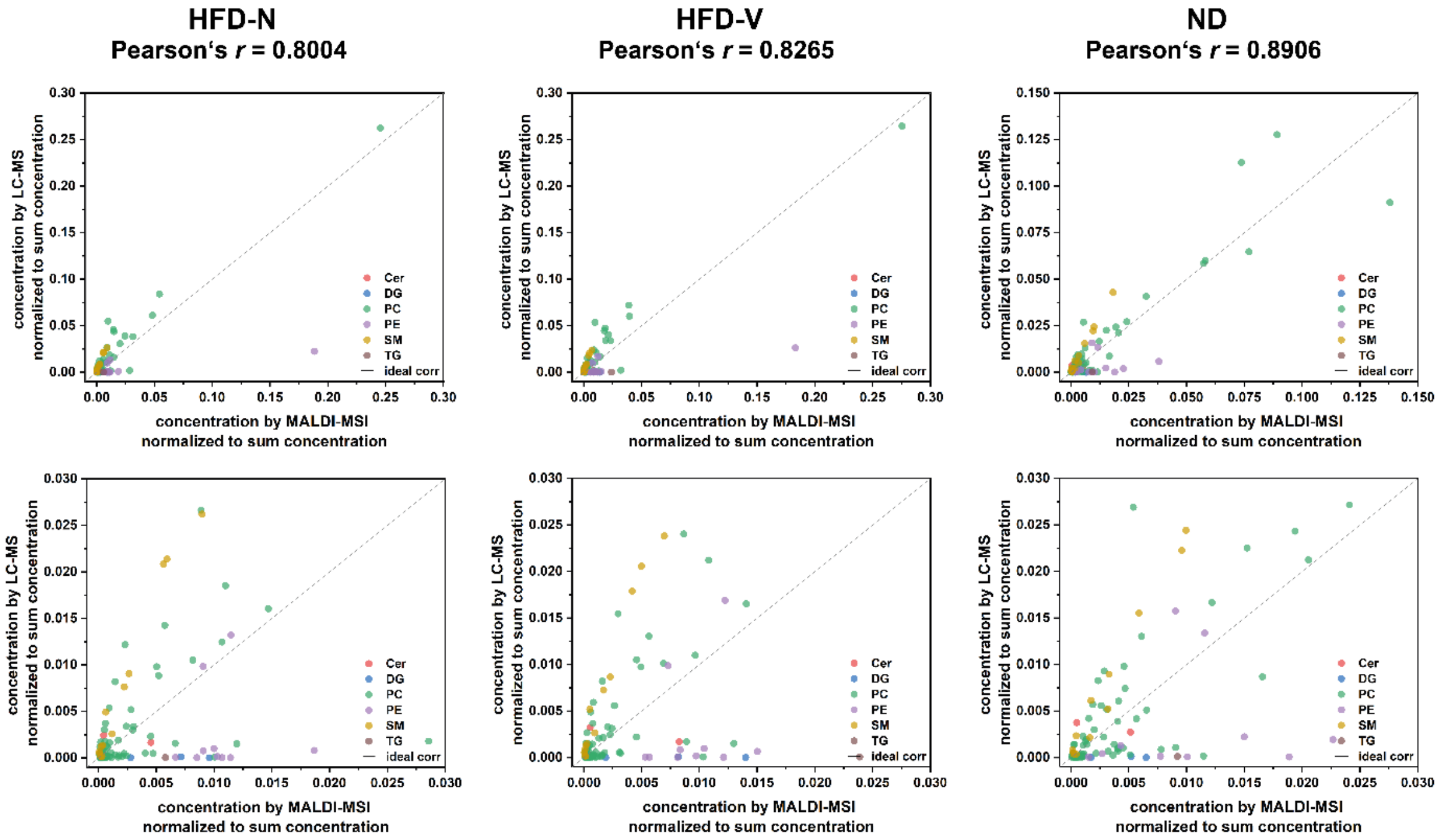

Figure S10. Pearson correlation between lipid concentrations determined by RP-LC-MS/MS and MALDI-MSI for mouse kidney of HFD-N, HFD-V, and ND mice. Individual lipid concentrations are normalized to the sum concentration for better comparison of both modalities. The grey diagonal line indicates perfect correlation of the methods. Upper row: full range of data points, lower row: zoom-in.
